# An Integrated Testing Strategy and Online Tool for Assessing Skin Sensitization of Agrochemical Formulations

**DOI:** 10.3390/toxics12120936

**Published:** 2024-12-23

**Authors:** Hung-Lin Kan, Shan-Shan Wang, Chun-Lin Liao, Wei-Ren Tsai, Chia-Chi Wang, Chun-Wei Tung

**Affiliations:** 1 Institute of Biotechnology and Pharmaceutical Research, National Health Research Institutes, Miaoli 35053, Taiwan; hlkan0422@nhri.edu.tw (H.-L.K.); shanwang@nhri.edu.tw (S.-S.W.); 2 Ph.D. Program in Environmental and Occupational Medicine, College of Medicine, Kaohsiung Medical University and National Health Research Institutes, Kaohsiung 80756, Taiwan; 3 Agricultural Chemicals Research Institute, Ministry of Agriculture, Taichung 41358, Taiwan; clliao@acri.gov.tw (C.-L.L.); wrt@acri.gov.tw (W.-R.T.); 4 Department and Graduate Institute of Veterinary Medicine, School of Veterinary Medicine, National Taiwan University, Taipei 10617, Taiwan; 5 Graduate Institute of Data Science, College of Management, Taipei Medical University, Taipei 10675, Taiwan

**Keywords:** adverse outcome pathway, skin sensitization, machine learning, SkinSensDB, SkinSensPred, pesticides, 3R

## Abstract

Non-animal assessment of skin sensitization is a global trend. Recently, scientific efforts have been focused on the integration of multiple evidence for decision making with the publication of OECD Guideline No. 497 for defined approaches to skin sensitization. The integrated testing strategy (ITS) methods reported by the guideline integrates in chemico, in vitro, and in silico testing to assess both hazard and potency of skin sensitization. The incorporation of in silico methods achieved comparable performance with fewer experiments compared to the traditional two-out-of-three (2o3) method. However, the direct application of current ITSs to agrochemicals can be problematic due to the lack of agrochemicals in the training data of the incorporated in silico methods. To address the issue, we present ITS-SkinSensPred 2.0 for agrochemicals and agrochemical formulations using a reconfigured in silico model SkinSensPred for pesticides. Compared to ITSv2, the proposed ITS-SkinSensPred 2.0 achieved an 11% and 16% improvement in the accuracy and correct classification rate for hazard identification and potency classification, respectively. In addition, an online ITS tool was implemented and available on the SkinSensDB website. The tool is expected to be useful for evaluating skin sensitization of substances.

## 1. Introduction

Skin sensitization is a key focus of chemical safety assessment [1,2]. Based on the 3Rs (replacement, reduction, and refinement) principle and adverse outcome pathway (AOP) for skin sensitization, non-animal alternative testing has become the preferred method for assessing skin sensitization of chemicals [3,4,5]. The Organization for Economic Co-operation and Development (OECD) has endorsed alternative methods to test skin sensitizers, including Test No. 442C [6], Test No. 442D [7], and Test No. 442E [8] for key events (KE1, KE2, and KE3, respectively). In addition, in silico models provide useful tools for assessing skin sensitization potential based on structure information [9,10,11].

Since each AOP-based assay addresses only one specific KE, the integrated analysis of evidence from various assays is crucial to improve regulatory acceptance. Efforts have been made to develop integrated testing strategies (ITSs) to combine multiple test results for better decision making [12,13,14]. The OECD has published defined approaches (DAs) for skin sensitization, including the 2o3 strategy, ITSv1, and ITSv2 [15,16]. The 2o3 strategy was based on two concordant results from the direct peptide reactivity assay (DPRA), KeratinoSens, or human cell line activation test (h-CLAT) to classify skin sensitization hazards. ITSv1 and ITSv2 incorporated h-CLAT, DPRA, and in silico hazard classification predictions, utilizing Derek Nexus and QSAR Toolbox, respectively. By replacing the KeratinoSens with in silico models, the ITS methods showed comparable performance at a much lower cost. These approaches integrated assay results of KEs and computer-based methods to analyze skin sensitization hazard and potency of chemicals with promising results in categorizing chemicals and agrochemical formulations [17]. In addition to the three ITSs, ITS-SkinSensPred [18] was recently proposed by replacing the in silico model with SkinSensPred [19] and showed comparable or improved performance for hazard identification and potency classification compared to ITSv1 and ITSv2. SkinSensPred is an AOP-aware transfer learning-based model for predicting human skin sensitizers. Through simultaneous learning of the task-specific knowledge and shared knowledge of four tasks of protein binding, keratinocyte activation, activation of dendritic cells, and human skin sensitization, the prediction accuracy was significantly improved.

The ITS-SkinSensPred tool performed well in categorizing chemicals, but its application to agrochemical formulations still needs improvement. This challenge primarily stems from the lack of agrochemical data in the training sets of current in silico tools [20,21,22,23]. Although AOP-based computational methods showed potential to alternate traditional animal testing, most existing data focus on general chemicals, pharmaceuticals, and cosmetics [24,25,26,27]. Current data on skin sensitization associated with pesticides are mainly derived from animal testing studies. However, the outcomes of these studies may not accurately reflect how humans respond, as predicted by existing AOP-based tools. There is a need to validate and interpret the predictive results and determine how to integrate the tools for regulatory use. Therefore, we reconfigured the applicability domain (AD) of SkinSensPred for agrochemical formulations, specifically targeting pesticides, such as herbicides, fungicides, and insecticides, in our previous study [28] to address the issues of species difference and distinct properties of pesticides. In brief, exclusion rules for chemicals outside the reconfigured AD for pesticides were derived using a decision-tree-based algorithm to define the consensus chemical space with matched animal testing data and predicted human responses for nonsensitizers [29,30]. The independent test results concluded a promising specificity for pesticide nonsensitizers.

We present ITS-SkinSensPred 2.0, an advanced tool combining the ITS methodology with SkinSensPred and a consensus chemical space for pesticide nonsensitizers. This new version effectively evaluates the skin sensitization potential of agrochemical formulations, demonstrating improved performance in hazard identification and potency classification. The integration of the reconfigured SkinSensPred for pesticides significantly enhances model coverage and prediction reliability. Additionally, a user-friendly ITS web tool was implemented, allowing users to input chemical data along with in vitro and in chemico test results to analyze the skin sensitization hazard and potency of various chemicals and formulations. ITS-SkinSensPred 2.0 represents great potential as an easily accessible and efficient tool for assessing skin sensitization, which is valuable in decreasing dependence on animal testing.

## 2. Materials and Methods

### 2.1. Dataset

A total of 27 agrochemical formulations were collected from a previous study [17]. Among the formulations, 15 were nonsensitizers (Globally Harmonized System of Classification and Labelling of Chemicals (GHS) Not Classified), 11 were weak or moderate sensitizers (GHS Category 1B), and 1 was a strong sensitizer (GHS Category 1A). The testing data of DPRA and h-CLAT were also collected from the study. The formulations may contain one or more active ingredients, and the in silico predictions for the formulation were based on the active ingredients. To be precise, if one of the active ingredients was positive and the concentration of that positive ingredient was at least 0.1% (*w*/*w*) in the formulation, then the in silico prediction for this formulation was considered a sensitizer. The predictions for the formulation will be considered inconclusive if it contains ingredients that cannot be predicted and all other predictions are negative; otherwise, the prediction be classified as negative. For a detailed list of the 27 formulations and their corresponding formation type, active ingredients, DPRA and h-CLAT scores derived from the ITS approach [31], and GHS classifications, please refer to Table S1.

### 2.2. The Reconfigured SkinSensPred for Pesticide Nonsensitizers

SkinSensPred is an online tool for predicting skin sensitization hazards [19]. It used a multitask extratree algorithm, an advanced machine learning approach that extends the original single-task-based extratree algorithm to learn shared knowledge from multiple learning tasks. In addition to the ordinary tree-splitting criteria, it introduced a task-splitting node to divide the whole dataset into task-specific datasets for learning task-specific knowledge. Its prediction accuracy was largely improved by simultaneously learning four tasks: protein binding, keratinocyte activation, dendritic cell activation, and human skin sensitization. The model was trained using data from SkinSensDB [32], a curated database for skin sensitization assays available at https://cwtung.nhri.edu.tw/skinsensdb (accessed on 1 July 2024). The SkinSensPred web tool is available at https://cwtung.nhri.edu.tw/skinsensdb/predict (accessed on 1 July 2024). Each test chemical was represented as a simplified molecular-input line-entry system (SMILES) string for in silico prediction. The webserver returned the prediction score for skin sensitization and applicability domain information for each input SMILES string. Compared to the original SkinSensPred aiming to predict human skin sensitizers, the reconfigured SkinSensPred with an applicability domain specific for pesticides can identify nonsensitizers with high concordance to animal testing data.

The applicability domain for the pesticide of SkinSensPred is a ruleset of structure characteristics represented by a graph fingerprint [33,34]. The ruleset was determined by applying a machine learning algorithm of the classification and regression tree (CART). Specifically, the animal testing data included three types of pesticides, herbicides, fungicides, and insecticides, which were utilized to predict human response. The structural features showing conflict results between SkinSensPred prediction and animal testing data were extracted and utilized as exclusion rules for identifying chemicals outside of the consensus chemical space. Our previous study showed that chemicals with a SkinSensPred score equal to or less than 0.4 and within the applicability domain for pesticides can be reliably predicted as nonsensitizers with a high concordance of 100% [28]. This study only considered the predictions within the applicability domain for reliable prediction of pesticide nonsensitizers.

### 2.3. ITS-SkinSensPred 2.0

Integrated testing strategy (ITS) approaches are score-based systems that address the KE1 of the AOP using a DPRA assay and KE3 using h-CLAT and incorporate an in silico tool for scoring. ITS-v1, ITS-v2, and ITS-SkinSensPred utilized different in silico tools to make the in silico evaluation of the formulation. The abovementioned three ITSs utilized in silico tools that were not specifically designed for agrochemicals where the majority of their training sets are general chemicals, pharmaceuticals, and cosmetics. To enable the capability for assessing skin sensitization potentials of agrochemical formulations, this work incorporated the reconfigured SkinSensPred for pesticides [28] to develop a new ITS-SkinSensPred 2.0 for agrochemical formulations.

In brief, the proposed ITS-SkinSensPred 2.0 is based on the same ITS scoring methods of ITS-SkinSensPred with an additional option to choose the applicability domains of SkinSensPred for agrochemicals and non-agrochemicals, which means the design of ITS-SkinSensPred 2.0 can handle both agrochemicals and non-agrochemicals. The ITS scoring process is a sum of scores from the DPRA assay results based on mean percent depletion for the cysteine and lysine peptides, h-CLAT outcomes based on the minimum induction threshold, and in silico skin sensitization hazard prediction. For each of the DPRA and h-CLAT assays, a score ranging from 0 to 3 was given according to the corresponding assay results. In addition, a binary score was assigned to the sensitizer and nonsensitizer according to in silico prediction. To conclude the in silico results, a sensitizing formulation is characterized by the presence of at least one sensitizer ingredient exceeding a concentration of 0.1%. On the other hand, the prediction is considered negative, except for formulations with any ingredients with unclear predictions or undefined structures that are categorized as inconclusive. The three scores were then summed into a final score [17].

For hazard identification, a chemical with a combined score of 2 to 7 was classified as a skin sensitizer. In contrast, a combined score less than or equal to 1 suggested a nonsensitizer. For potency classification, a combined score of 6 to 7 was classified as Category 1A, and a combined score of 2 to 5 was classified as Category 1B. The rest were not classified (NC). In some cases, the inconclusive prediction, especially the potency classification, may be assigned when one input is unavailable or out of the domain.

### 2.4. Implementation of an Online Tool of ITS-SkinSensPred 2.0

To make ITS-SkinSensPred 2.0 more useful, a user-friendly web tool has been developed. This tool utilizes a range of technologies, including HTML (HyperText Markup Language), PHP, JavaScript, Java, and R, to ensure functionality and accessibility. The web tool was hosted in a Ubuntu-based system with the Apache HTTP Server, providing a stable and reliable environment for users. The ITS-SkinSensPred 2.0 user interface was created to streamline the process of identifying hazards and determining potency by automatically scoring the DPRA and h-CLAT results entered by the user, obtaining the in silico prediction, and concluding the final results based on the three components. For the in silico prediction, users can indicate whether the evaluated chemical is a pesticide or not, and the prediction results will be automatically determined by applying corresponding applicability domains. The interface will then show the results of hazard classification (e.g., sensitizers and nonsensitizers) and potency prediction (e.g., Category 1A or 1B).

### 2.5. Performance

The performance of ITS-SkinSensPred 2.0 for hazard identification was evaluated by sensitivity (true positive rate), specificity (true negative rate), accuracy, and balanced accuracy. For potency classification, accuracy was calculated for each subcategory and overall accuracy was calculated for all chemicals. The measurements were defined as follows,

(1) $$Sensitivity=\frac{TP}{TP+FN},$$

(2) $$Specificity=\frac{TN}{TN+FP},$$

(3) $$Accuracy=\frac{TP+TN}{TP+TN+FP+FN},$$

(4) $$Balanced\;accuracy=\frac{Sensitivity+Specificity}{2},$$

(5) $$Coverage=\frac{Number\;of\;agrochemical\;formulations\;for\;which\;a\;prediction\;could\;be\;made}{Total\;number\;of\;agrochemical\;formulations\;tested},$$

where TP, TN, FP, and FN are the numbers of true positives, true negatives, false positives, and false negatives, respectively, which were calculated by comparing the results of prediction and experimental data.

## 3. Results and Discussion

### 3.1. Hazard Identification

ITS-SkinSensPred 2.0 was conducted to predict the dataset of agrochemical formulations for hazard identification and compared with the published methods of 2o3, ITSv2, and ITS-SkinSensPred. The assessment results are shown in Table 1 and Table S1. The reference dataset comprised 27 agrochemical formulations, of which 44% (12/27) were identified as sensitizers in animal testing. The 2o3 method based on three assays of DPRA, KeratinoSens, and h-CLAT showed the best performance with a sensitivity of 90%, a specificity of 67%, an accuracy of 79%, and a balanced accuracy of 78%, respectively. However, its coverage of 70% is relatively low. Although the 2o3 strategy is effective, the experiments necessary for reaching a final decision are resource intensive.

To maximize the use of laboratory data, ITS scoring methods were proposed for both hazard identification and potency classification, considering assays from KE1, KE3, and in silico methods. ITSv2 with a high coverage of 89% showed good performance with a sensitivity of 91% but a very low specificity of 23%, leading to worst accuracy and balanced accuracy of 54% and 57%, respectively. ITS-SkinSensPred is associated with a relatively low coverage of 63%, which is expected due to the lack of pesticides in its training dataset. Nevertheless, the incorporation of SkinSensPred provides better accuracy and balanced accuracy of 59% and 57%, respectively. In contrast, the proposed ITS-SkinSensPred 2.0 with a high coverage of 74% provides superior performance over the two ITSs with accuracy and balanced accuracy of 65% and 67%, respectively. The detailed in silico prediction scores, the final ITS scores, and the GHS classification of ITS-SkinSensPred 2.0 for each formulation are given in Table S1. A high 89% sensitivity was obtained for ITS-SkinSensPred 2.0, which is comparable with all other methods, and a good 45% specificity was obtained for ITS-SkinSensPred 2.0, which is much better than the other two ITSs. This improved specificity is due to the integration of the reconfigured applicability domain (AD) for pesticides. In our previous study, chemicals with a SkinSensPred score of 0.4 or lower that fell within the applicability domain for pesticides can be reliably identified as nonsensitizers [28]. The application of ITS-SkinSensPred 2.0 to hazard identification for agrochemical formulations showed a 22%, 11%, and 10% improvement over ITSv2 in terms of specificity, accuracy, and balanced accuracy, respectively. ITS-SkinSensPred 2.0 also showed a 20%, 6%, and 10% improvement over ITS-SkinSensPred in terms of specificity, accuracy, and balanced accuracy, respectively. Overall, ITS-SkinSensPred 2.0 presented high specificity for identifying pesticide nonsensitizers while still maintaining effective sensitivity performance.

### 3.2. Potency Classification

The evaluation results for potency classification are shown in Table 2 and Table S1. In the context of potency classification, low underpredicted rates of 4%, 7%, and 6% were obtained for all the compared methods of ITSv2, ITS-SkinSensPred, and ITS-SkinSensPred 2.0, respectively. ITSv2 with a high coverage of 85% showed the lowest correct classification of 43%. With a high overpredicted rate of 52% for ITSv2, this conservative approach poses no harm in ensuring safety in regulatory settings but may result in additional costs for the prevention of potential hazards. ITS-SkinSensPred showed improved performance with a correct classification rate of 53% and an overpredicted rate of 40%, respectively. It exhibits a 10% enhancement in the correct classification rate and a 12% reduction in the overpredicted rate. The proposed model with a reduced overprediction rate and a comparable underprediction rate compared to the existing methods provides a better balance between safety and economics. However, due to the low coverage of SkinSensPred for pesticides, the coverage of ITS-SkinSensPred is only 56%.

The proposed ITS-SkinSensPred 2.0 showcases remarkable improvement over ITSv2 and ITS-SkinSensPred regarding correct classification and overpredicted rates. With a correct classification rate of 59% and an overpredicted rate of 35%, ITS-SkinSensPred 2.0 shows a 16% increase in the correct classification rate and a 17% decrease in the overpredicted rate compared to ITSv2. A 6% and 5% improvement in the correct classification and overpredicted rates were obtained when comparing ITS-SkinSensPred 2.0 to ITS-SkinSensPred. The coverage of ITS-SkinSensPred 2.0 is 63%, which is better than ITS-SkinSensPred (56%) but worse than ITSv2 (85%). Overall, ITS-SkinSensPred 2.0 using the reconfigured SkinSensPred for in silico prediction of pesticide nonsensitizers notably enhanced the performance and expanded the applicability of ITS methods to skin sensitization.

Since the reconfigured SkinSensPred is applicable only for pesticide nonsensitizers, a moderate coverage of ITS-SkinSensPred 2.0 is expected. Also, the high specificity of the reconfigured SkinSensPred for pesticide nonsensitizers led to a largely reduced overpredicted rate compared to ITSv2 and ITS-SkinSensPred. While the issue of insufficient pesticide information for training computational models [35] remains, the reconfiguration of current in silico methods can still be useful in the setting of ITS.

### 3.3. ITS-SkinSensPred 2.0 Web Tool

**Figure 1:**
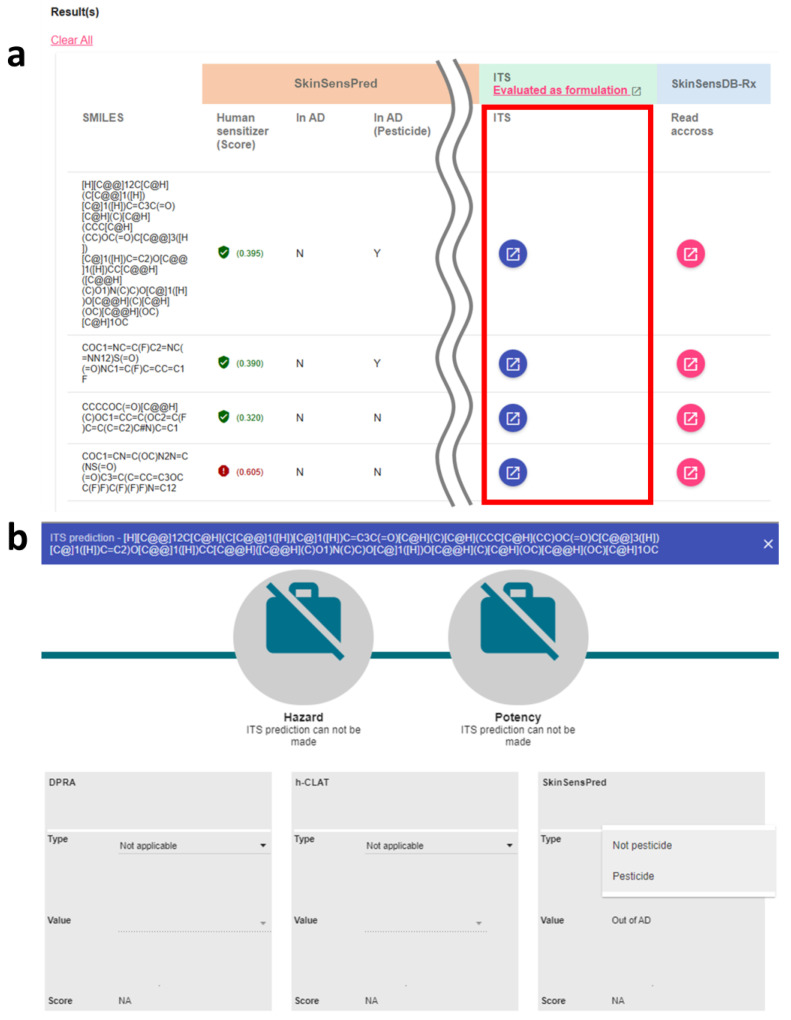
ITS-SkinSensPred 2.0 user interface for evaluating single chemicals. (**a**) Result table of each SkinSensPred prediction with original applicability domain (AD) and AD for pesticides and the ITS tool shown in the red frame, (**b**) interactive user interface for calculating the ITS-SkinSensPred 2.0 score. Users can input chemical structure information to predict skin sensitizers and utilize the ITS tool to analyze agrochemicals and other chemicals.

The ITS-SkinSensPred 2.0 platform has been designed to facilitate the integrated testing strategy (ITS) for both agrochemicals and non-agrochemicals and their formulations by automating the scoring for DPRA, h-CLAT, and in silico prediction obtained from SkinSensPred. This web-based interface integrates seamlessly with the SkinSensPred tool with two schemes for agrochemicals and chemicals other than agrochemicals. As shown in Figure 1, the user-supplied SMILES of chemicals will be automatically converted to feature vectors and fed into the SkinSensPred model to generate prediction scores, model applicability for non-pesticide chemicals and pesticides, and structure alerts. The developed ITS-SkinSensPred 2.0 tool can be accessed via the ITS column.

**Figure 2:**
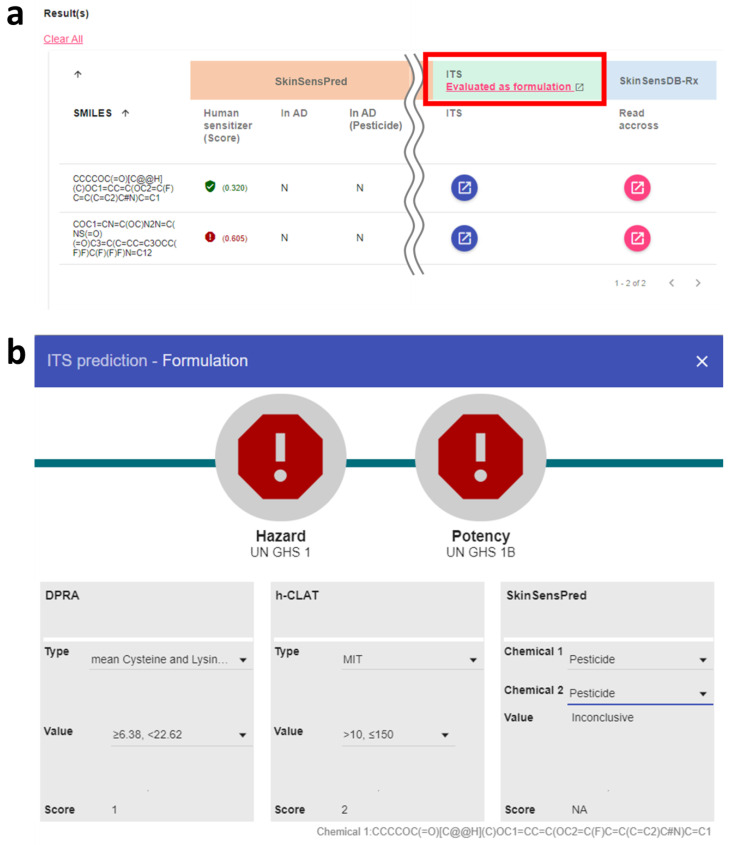
ITS-SkinSensPred 2.0 user interface for evaluating formulations. (**a**) Result table of SkinSensPred prediction for each component of a formulation and the ITS tool for evaluating the formulation shown in the red frame, (**b**) interactive user interface for calculating the ITS-SkinSensPred 2.0 score. Users can evaluate the components as a formulation by clicking the “evaluated as formulation” link and choosing the corresponding types of chemicals in the interactive user interface to derive the final score.

Two modes of ITS assessment were available for single chemicals and formulations with multiple chemicals, as shown in Figure 1a and Figure 2a, respectively. Users can choose to evaluate a single ingredient or a formulation with multiple chemicals listed in the result table. For the evaluation of a single chemical, users can click the corresponding blue button in the ITS column of the input chemical (Figure 1a) to gain access to the interactive interface for ITS evaluation of the skin sensitization hazard and potency of the test chemical. As for the evaluation of skin sensitization of formulations, users can click the link in the header of the ITS column (Figure 2a) to access the interactive interface for an ITS evaluation of the listed chemicals comprising a formulation.

The interactive interface, displayed in Figure 1b and Figure 2b, provides a real-time evaluation in response to the user-supplied experimental data type and value levels of DPRA and h-CLAT. For the panels of DPRA and h-CLAT, users have to select the corresponding type and value of the experiments using the dropdown menus, as shown in Figure 1b. The dropdown menu options and their corresponding scores are determined based on the ITS scoring scheme [15]. When selecting the options of type and value in each panel of DPRA and h-CLAT, the corresponding score will be automatically calculated and shown at the bottom of the panel, as shown in Figure 2b. The left and right upper icons showed the final results of hazard identification and potency prediction. The red and green icons indicate the ITS classification results of potential skin sensitizer and not classified, respectively, with the corresponding classification results below the icons.

As for the scoring of in silico predictions, predictions were automatically conducted with the applicability domain for the user-supplied chemical type of “pesticide” or “not pesticide” to ensure a more accurate domain-specific estimation of model applicability. To evaluate a single chemical, its SMILES string will be shown in the title of the ITS panel (Figure 1b). As for the formulation, the SMILES information for the mouse-hovered chemical will be displayed in the lower right corner (Figure 2b). This automated system will present hazard and potency evaluation outcomes while any missing or inapplicable data will be displayed to help streamline the regulatory decision-making process.

### 3.4. Case Study Based on ITS-SkinSensPred 2.0

To illustrate the capabilities of the ITS tool, several case studies were conducted and analyzed. For a detailed list of the 27 formulations, please refer to Table S1. First, we focused on agrochemical formulations that were previously classified as “Not Classified” in in vivo GHS classification, such as Florasulam (formulation 2 in the list) and Spinosad (formulation 18 in the list). These formulations had negative results in DPRA, borderline results in KeratinoSens, and positive results in h-CLAT, which led to inconclusive outcomes under the 2o3 DA [17]. The 2o3 approach is an effective method that has shown strong sensitivity, specificity, and overall accuracy in predicting the skin sensitization potential of chemicals. However, the reactive assays might potentially lead to “false positive” results, such as DPRA, which, for example, can produce false positives due to peptide dimers, cysteine oxidation, or non-covalent peptide binding [36]. Similarly, the activation of the ARE-Nrf2 pathway in the KeratinoSens assay may not be directly related to skin sensitization processes, which could lead to overestimations [37]. h-CLAT also faces challenges, as dendritic cell activation observed in the assay may not always correlate with skin sensitization, and its reliability for substances with a log Kow >3.5 or mixtures remains limited [17,38].

**Figure 3:**
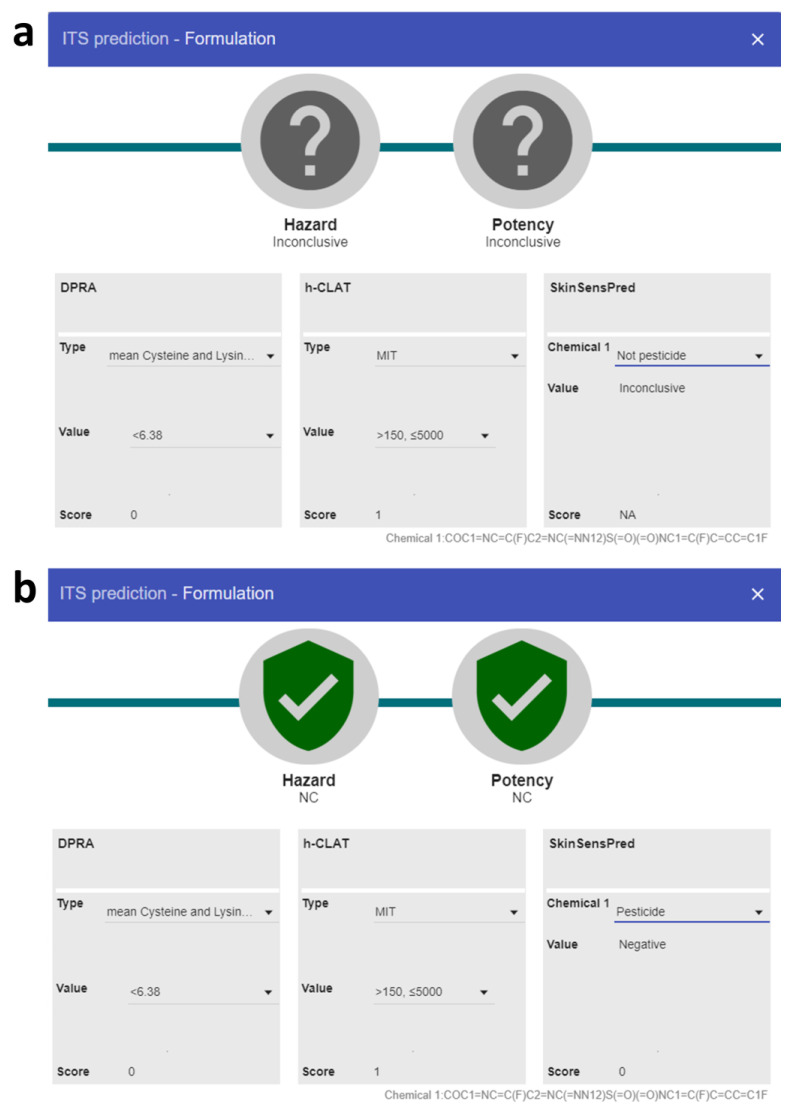
The ITS analysis results for Florasulam (formulation 2) using (**a**) ITS-SkinSensPred and (**b**) ITS-SkinSensPred 2.0.

Although the ITS strategy incorporates multiple assays and utilizes in silico models to enhance decision making, some limitations still exist. For the two agrochemical formulations, the scoring system showed that these two formulations had a score of 0 in DPRA (which means they showed a mean cysteine and lysine depletion of less than 6.38%) and a score of 1 in h-CLAT (meaning the minimum induction threshold is between 150 and 5000). In this case, in silico tools play a decisive role. According to the previous study [17], ITSv2 identified these formulations as GHS 1B, which was an overpredicted result. ITS-SkinSensPred with default settings, although improving specificity and accuracy from ITSv2, was still unable to effectively address these cases and was finalized with an inconclusive prediction (Figure 3a). In contrast, by choosing the type of chemical as a pesticide, ITS-SkinSensPred 2.0 demonstrated its reliability in predicting pesticide nonsensitizers and successfully identified formulations 2 and 18 as “Not Classified,” indicating that they are nonsensitizers (Figure 3b).

While overpredicted issues were reduced, the sensitivity for identifying skin sensitizers is still good. In the case of formulations 3, 12, 15, 21, and 27, ITS-SkinSensPred 2.0 successfully assigned the formulations as GHS 1B. Although ITS-SkinSensPred 2.0 achieved good performance on both sensitizers and nonsensitizers, there are still some limitations to consider when using the tool. For example, the in silico model of SkinSensPred is not applicable for predicting inorganic compounds, metals, and pesticide skin sensitizers.

## 4. Conclusions

This study presents a novel ITS-SkinSensPred 2.0 to assess skin sensitization potential for agrochemical formulations with good performance in hazard identification and potency classification. The comparison to published ITS methods showed the great potential of ITS-SkinSensPred 2.0 for hazard identification and potency classification of agrochemical formulations with comparable or superior performance. The incorporation of reconfigured SkinSensPred for pesticides significantly improved model coverage and prediction reliability for agrochemical formulations. The user-friendly interactive interface for ITS-SkinSensPred 2.0 can provide real-time calculations of hazard and potency outcomes, streamlining regulatory decision making and reducing the need for manual calculations. While regulatory authorities can utilize the DASS App developed by To et al. [39] for skin sensitization assessments, the absence of ITS tools with automatic in silico prediction tools means users must spend extra time on data collection or searching for validated in silico tools. Overall, ITS-SkinSensPred 2.0 shows great potential as an easily accessible tool for skin sensitization assessment that is useful in reducing reliance on animal testing. Future work includes the collection of more skin sensitization data for other agrochemicals, including fertilizers, soil amendments, and additives, as well as tuning the prediction model and adjusting the applicability domain according to the collected data to expand the use of the proposed method. Further, ITS-SkinSensPred will be tested against more agrochemical formulations and agrochemical products with both active and other ingredients to inform regulatory use.

## Figures and Tables

**Table 1:** Performance of ITS methods for hazard identification of agrochemical formulations.

Method	Sensitivity (%)	Specificity (%)	Accuracy (%)	Balanced Accuracy (%)	Coverage (%)
**2o3 ^1^**	90	67	79	78	70
**ITSv2 ^1^**	**91**	23	54	57	**89**
**ITS-SkinSensPred ^2^**	89	25	59	57	63
**ITS-SkinSensPred 2.0 (this study)**	89	**45**	**65**	**67**	74

^1^ Data obtained from Strickland et al. [17]. ^2^ Data obtained from Wang et al. [18].

**Table 2:** Performance of ITS methods for GHS potency classification of agrochemical formulations.

Method	Correct Classification (%)	Underpredicted (%)	Overpredicted (%)	Coverage (%)
**ITSv2 ^1^**	43	**4**	52	**85**
**ITS-SkinSensPred ^2^**	53	7	40	56
**ITS-SkinSensPred 2.0 (this study)**	**59**	6	**35**	63

^1^ Data obtained from Strickland et al. [17]. ^2^ Data obtained from Wang et al. [18].

## Data Availability

All data used in this study are available in the Supplementary Materials. The data that support the findings of this study are available from the corresponding author upon reasonable request.

## Supplementary Materials

The following supporting information can be downloaded at https://www.mdpi.com/article/10.3390/toxics12120936/s1, Table S1: The ITS-SkinSensPred 2.0 (integrated testing strategy) results for 27 agrochemical formulations.
